# Associations between diet, gut microbiota and markers of CRC risk

**DOI:** 10.1186/2049-3002-2-S1-P45

**Published:** 2014-05-28

**Authors:** Tyler Culpepper, Maria Ukhanova, Xiaoyu Wang, Yijun Sun, Volker Mai

**Affiliations:** 1 Epidemiology, Univ. of Florida, Gainesville, FL, USA; 2 Microbiology, State Univ. of NY, Buffalo, NY, USA

## Background

Recent advances in our understanding of the contributions of gut microbiota to host metabolism have renewed research interest in how distortions in microbiota activities correlate with health status. While extensive microbiota surveys have already revealed the complexity of microbiota dynamics there is a need to better understand mechanisms through which microbiota can affect host metabolism. We have recently shown that the amount of energy available to the host after intake of resistant maltodextrin varies among individuals and is associated with microbiota. Here we hypothesized that microbiota i) is associated with dietary habits, ii) correlates with colorectal cancer (CRC) risk and iii) can affect the methylation status in nearby gut epithelium, which might be a mechanism contributing to colorectal carcinogenesis.

## Materials and methods

In 126 human volunteers undergoing a screening colonoscopy we determined associations between dietary intake, gut microbiota composition, epithelial methylation status and polyp prevalence. We determined: i) dietary habits using 3-day food records, FFQ (Block 98), and Meat Module Questionnaire; ii) medical history , iii) microbiota composition in a fecal sample collected before and multiple biopsy samples collected during the colonoscopy, during which we determined iv) polyp status. Microbiota composition was analyzed in DNA extracted from stool and biopsy samples using a 16S rRNA based approach. Sequence reads were binned into operational taxonomic units (OTUs) using ESPRIT and analyzed using QIIIME. We then performed a discriminant analysis to identify a microbiota pattern associated with polyp prevalence. For a subset of fecal samples a shotgun metagenomic analysis was undertaken. An exploratory methylation analysis was performed in DNA extracted from 12 biopsy samples using the Infinium HumanMethylation450 beadchip.

## Results

Various microbiota associations with dietary intake as well as differences in the presence of OTUs between subjects with polyps and controls (p<0.01) were observed, with the most significant of these differences detected in subjects with high risk polyps. A predictive model based on 27 OTUs was effective in identifying subjects with at least one polyp (AUC =0.81). Butyrate producing bacteria appeared decreased among polyp cases. Methylation status at multiple sites was associated with polyp status, which correlated with specific microbiota differences.

## Conclusions

Our observations confirm microbiota contributions to host metabolism. Larger studies in targeted populations will be needed to determine more details of the complex mechanisms of interactions between microbiota activities and host metabolism.

